# Substance abuse in first-episode psychosis at Chris Hani Baragwanath Hospital

**DOI:** 10.4102/sajpsychiatry.v32i0.2542

**Published:** 2026-01-19

**Authors:** Precious N. Shandu, Yumna Minty

**Affiliations:** 1 Department of Psychiatry, Faculty of Health Sciences, University of the Witwatersrand, Johannesburg, South Africa

**Keywords:** first-episode psychosis, early intervention psychiatry, cannabis use, methamphetamine use, substance use disorder, risk factors, psychiatric comorbidities

## Abstract

**Background:**

In South Africa, the use of substances is associated with serious health challenges, exacerbated by limited health resources. Substance use is often associated with first-episode psychosis (FEP). Prevention and treatment protocols can be established by understanding the socio-demographic and clinical characteristics of patients with FEP.

**Aim:**

To determine the prevalence, patterns, socio-demographic and clinical factors associated with substance use in patients with FEP at Chris Hani Baragwanath Academic Hospital.

**Setting:**

The study was conducted at Chris Hani Baragwanath Academic Hospital, a tertiary healthcare facility in Soweto, South Africa.

**Methods:**

The study was a retrospective chart review analysis of clinical records of 200 patients presenting with FEP. The data were then statistically analysed, and patients with and without substance use in FEP were compared.

**Results:**

The prevalence of substance use was 73.6%. Most patients were male and between 21 years old and 30 years old (37.0%). Substance-induced psychotic disorder was the most common diagnosis. Cannabis (THC) (46.0%) was the most commonly used substance. Substance use was associated with aggression (45.0%), and only 34.0% of substance users were referred to social services.

**Conclusion:**

Substance use is a modifiable risk factor in the presentation of psychotic disorders. Integrated models of care, addressing both mental health and substance use and targeting early intervention, are essential to improve patient outcomes.

**Contribution:**

In this study, the focus was on the interrelationship between substance use and psychosis. It emphasised the need for integrated strategies for the treatment of mental health and substance use. It provides a crucial insight into the socio-demographic and clinical predictors of substance use in patients with FEP, which can inform clinical practice and intervention strategies.

## Introduction

Psychotic disorders are a group of disorders that affect a person’s thoughts and perceptions, and make it difficult for them to recognise what is real and what is not.^1^ It is defined by positive symptoms (hallucinations, delusions, disorganised speech and behaviour), negative symptoms (reduced emotional expression, a lack of motivation and social withdrawal) and cognitive decline.^1^ Psychotic disorders can be differentiated into delusional disorder, brief psychotic disorder, schizophreniform disorder, schizophrenia, substance or medication-induced psychotic disorder, psychotic disorder because of another medical condition and schizoaffective disorder.^2^ First-episode psychosis (FEP) is a term used to describe an individual’s first presentation to a healthcare facility with psychotic symptoms, with no prior treatment for psychosis.^3^ The causes of psychosis are multifactorial, and it is often difficult to determine the precise aetiology of FEP.^4,5^ However, studies have shown that a combination of biological and genetic variables can predispose someone to psychotic symptoms.^4,6^ For example, when these individuals are exposed to occurrences such as stressful circumstances or substances, they may experience a psychotic episode.^4^

Psychosis is a common presentation in individuals who use substances.^7^ Barnet et al. showed that the overall prevalence of substance use in patients with FEP was double that in the general population, meaning that twice as many with psychosis use substances as the general population.^8^ According to the World Health Organization (WHO), 1.3% of the total burden of mental health disorders stems from the use of psychoactive substances, and it is estimated that about 39.5 million people are affected by substance use disorder globally.^9^ A study done at Lentegeur Psychiatric Hospital in the Western Cape showed that more than 90.0% of patients admitted to the acute admission ward presented with a psychotic disorder, and 28.0% of those patients had presented with FEP; in addition, 62.0% of the general acute admissions had used substances prior to their admission.^10^ Di Fort et al. in their multicentre review found that the use of substances often preceded the onset of psychosis and that patients who had presented with FEP had most likely used cannabis for at least 5 years prior to symptoms onset.^5^

In South Africa, the use of substances in individuals with mental illness is a growing concern.^11^ Comorbid substance use is common in patients with FEP.^10,12,13^ A study done at Dora Nginza Hospital in the Eastern Cape showed that patients presenting with FEP had a high prevalence of substance use, with cannabis being one of the most commonly used.^12^ A study done in Johannesburg by Anic and Robertson found a 67% prevalence of substance use in patients admitted to an acute psychiatric ward.^14^

Psychotic symptoms can occur acutely during intoxication and/or during withdrawal. They can also occur in the longer term, where the symptoms are ongoing despite no recent use of a substance.^15^ Research that have studied the effects of comorbid substance use in patients already diagnosed with a psychotic disorder showed that they often present with severe physical and verbal aggression, damage to property, an increase in suicidal and criminal activity and are most likely to be brought to the hospital by police officers or ambulance officials.^14,16^ These patients are more likely to commit violent crimes,^17^ and violence has been found to be a major factor leading to patients requiring inpatient involuntary admission.^10,16^

Policies have been formulated to address the problem of substance abuse in the country, such as the National Drug Master Plan 2019–2024.^18^ However, the gap between enacted and implemented policies is enormous, and as a result, more intervention is required to address the problem.^19^ Substance use is a modifiable risk factor for both the onset of psychosis and worse outcomes in psychotic disorders,^20^ and early aggressive interventions are essential to improve the prognosis.

While the burden of substance use and mental illness is increasing in South Africa,^12^ there is a shortage of healthcare provision.^11^ Understanding the factors associated with the use of substances in patients with FEP is crucial to the development of practical preventative guidelines and policies for these patients and an integrative approach to mental health management.^21^

This study aimed to determine the prevalence and patterns of substance use as well as associated factors in FEP patients at Chris Hani Baragwanath Academic Hospital (CHBAH). These findings will provide valuable information on the factors associated with the use of substances among FEP patients, guiding clinical management plans and resources to improve care for this population.

## Research methods and design

### Study design and setting

This study was a retrospective record review. A retrospective record review is a study design that allows the analysis of previously collected data from medical records.^22^ This design is particularly suitable in research done in a hospital, where prospective studies are not feasible.^22^ Using existing records allows researchers to answer clinical questions, analyse trends and associations, and look at utilisation of resources and patient outcomes.^22^ The study period was limited to 2 years, 2022 to 2023; this was to guarantee that there were enough patients with and without substance use to make a significant comparison. This study was conducted within the Department of Psychiatry at CHBAH, located in Soweto, Johannesburg (JHB), South Africa. Johannesburg is the largest city located in the centre of the heavily populated and economically important Gauteng province.^23^ Chris Hani Baragwanath Academic Hospital is the third largest health facility globally with a total bed capacity of approximately 3200.^22^ The Department of Psychiatry runs a 165-bed unit with four adult wards (two male and two female) as well as a child and adolescent ward.^24^ The psychiatric unit operates at almost full capacity, with an average of 15 daily admissions and a bed occupancy rate of about 90%.^24^ The population served by CHBAH is predominantly black and underprivileged, with an estimated 40% of the population being unemployed.^25^

### Study population and sampling strategy

The sample size was calculated using sensitivity and specificity likelihood ratios. For sensitivity, the formula used was: *n* = *Z*2*Se(1-Se)/*d*2*P, where *n* = sample size, *Z* = *Z* statistic for confidence level, Se = Assumed sensitivity, *d* = Precision and *P* = Prevalence. For specificity, the formula used was: *n* = *Z*2*Sp (1-Sp)/*d*2*P, where *n* = sample size, *Z* = *Z* statistics for a level of confidence (1.96 for 95% confidence level), Sp = Assumed specificity, *d* = Precision and *P* = Prevalence.

With sensitivity and specificity at 95%, precision at 10% and the prevalence at 17.4% (based on Franken et al.),^10^ a sample size of 105 patients per group using or not using substances was deemed to be adequate as per the sensitivity formula. The specificity formula generated a size of 59 patients per group. The average of the two (82) was assessed as appropriate for this study. Hence, 100 patients who used substances and 100 patients who did not were selected for the study.

The inclusion criteria were as follows:

- Medical records that documented all the relevant information needed for the study
- Patients aged 14 years and older
- Patients admitted during the periods of 2022 and 2023
- Patients diagnosed with a psychotic disorder for the first time.

The exclusion criteria were as follows:

- Patients with psychotic symptoms, who met the Diagnostic and Statistical Manual of Mental Disorders, Fifth Edition (DSM-5) criteria for a mood disorder (and thus the diagnosis would be a mood-spectrum disorder, rather than a psychotic disorder)
- Medical records that had incomplete relevant information were excluded.

### Data collection

The CHBAH Department of Psychiatry’s online database for discharge summaries was used as the source material. Discharge summaries from 01 January 2022 to 31 December 2023 were analysed, and the following data were extracted:

- The presence of a FEP
- Demographics (age, gender, marital status, employment status and length of hospital stay)
- Reason for presentation on admission (presence of aggression)
- The presence of substance use – from history or multidrug urine testing
- The type or types of substance used
- The psychiatric diagnosis
- Comorbid medical conditions
- Referral to a social worker for rehabilitation centre assistance.

Data were extracted into a predesigned Excel spreadsheet with preset columns for each variable. This ensured consistency of data collection.

### Data analysis

Statistical analyses were conducted in R software (version 4.0.0; www.R-project.org). Categorical data were reported as counts and percentages, while continuous data were reported as median and interquartile ranges (IQRs). Data were presented in charts, tables and text.

The prevalence of substance use in patients presenting with FEP at Chris Hani Baragwanath Academic Hospital was reported as a percentage of the total FEP in 2022 and 2023, with 95% confidence intervals (CIs). To compare socio-demographic and clinical variables of patients who used substances and those who did not, Pearson’s Chi-squared and Fisher’s exact tests were used to analyse categorical variables. The continuous variable, days of admission, was determined to be non-parametric (Shapiro-Wilk test) and was analysed using the Mann-Whitney U test for non-parametric data. All analyses were two-tailed, with model-level significance set at 0.05.

### Reliability and validity

All relevant data from official discharge summaries were extracted by the principal researcher from the Department of Psychiatry official online database. The systematic approach to extraction and input into the Excel worksheet enhanced the reliability.

### Ethical considerations

As this was a retrospective record review, patient consent was not required to access the data. Ethical approval to conduct this study was obtained from the Department of Psychiatry, Chris Hani Baragwanath Academic Hospital and the University of the Witwatersrand Human Research Ethics Committee (No. M240652). The study was also registered with the National Health Research Database (GP_202404_068). Data were collected in an entirely anonymous fashion, with no patient-identifying variables included.

## Results

### Prevalence of substance use

A total of 526 patients with FEP were admitted to CHBAH during 2022 and 2023. Of these, 387 patients used substances, 139 either did not use substances or were not recorded to have used substances. The prevalence of substance use in the study population was determined to be 73.6% (CIs: 68.8%, 77.3%).

### Socio-demographic characteristics of patients with first-episode psychosis

This study comprised a randomly selected subset of the 526 patients, including 100 patients who used substances and 100 patients who did not use substances.

The majority of patients included in this study were male (63.0%; Table 1). A significant number were 21–30 years old (37.0%), single (83.5%) and unemployed (74.5%; Table 1). The youngest and oldest patients identified were 15 years and 54 years old, respectively. Most patients were not aggressive (63.5%) and were not referred to a social worker (74.0%; Table 1). Patients spent a median of 24 days (1st IQR = 14 days; 3rd IQR = 39 days) in admission.

**TABLE 1:** Socio-demographic characteristics of patients with first-episode psychosis.

Variables	Count	%	*P*-value
**Gender**			*P* < 0.001
Female	74	37.0	-
Male	126	63.0	-
**Age (years)**			*P* < 0.001
< 20	45	22.5	-
21–30	74	37.0	-
31–40	47	23.5	-
41–50	21	10.5	-
> 50	13	6.5	-
**Marital status**			*P* < 0.001
Single	167	83.5	-
Married	30	15.0	-
Divorced	3	1.5	-
**Employment**			*P* < 0.001
Employed	26	13.0	-
Scholar	25	12.5	-
Unemployed	149	74.5	-
**Aggressive**			*P* < 0.001
Yes	73	36.5	-
No	127	63.5	-
**Referral to a social worker**			*P* < 0.001
Yes	52	26.0	-
No	148	74.0	-

Note: Youngest patient – 15 years old; oldest patient – 54 years old; *P*-value = Pearson’s Chi-squared tests; all tests were statistically significant.

### Clinical characteristics of patients with first-episode psychosis

A significantly greater number of patients were diagnosed with substance-induced psychotic disorder (SIPD) (40.0%), followed by patients with schizophrenia (23.5%) and psychotic disorder because of another medical condition (AMC) (18.0%) (*P* ≤ 0.001; Table 2). Most patients had no medical comorbidities (66.8%), which was significantly different from chance (i.e., a null model; *P* ≤ 0.001; Table 2). Most of those with a medical comorbidity had blood-borne infections (20.7%; Table 2).

**TABLE 2:** Clinical characteristics of first-episode psychosis patients (*n* = 200).

Variables	Count	%	*P*-value
**Psychiatric diagnosis**			*P* < 0.001
Brief psychotic disorder	22	11.0	-
Delusional disorder	3	1.5	-
Psychotic disorder because of AMC	36	18.0	-
Schizoaffective disorder	6	3.0	-
Schizophrenia	47	23.5	-
Schizophreniform	5	2.5	-
Substance-induced psychotic disorder	80	40.0	-
Unspecified psychotic disorder	1	0.5	-
**Medical comorbidities**			*P* < 0.001
Asthma	1	0.5	-
Nutritional deficiencies	6	2.8	-
Dyslipidaemia	2	1.0	-
Epilepsy	5	2.4	-
Blood-borne infections	43	20.7	-
Hypertension	8	3.8	-
Hypothyroidism	1	0.5	-
Thumb amputation-osteomyelitis	1	0.5	-
Traumatic brain injury	2	1.0	-
None	139	66.8	-

AMC, another medical condition.

### Substances used

All counts of substances used by the 100 patients in the substance-using group were considered in the analyses, resulting in a larger sample size, because 59% of the patients used more than one substance.

A significant majority took cannabis (46.0%), followed by methamphetamine (27.0%) and ethanol (12.7%) (*P* ≤ 0.001; Table 3). The prevalence of all other substances used was less than 13.0%.

**TABLE 3:** Substances used by patients with first-episode psychosis (*n* = 100).

Variable	Count	%
Cannabis (tetrahydrocannabinol)	87	46.0
Methamphetamine	51	27.0
Ethanol (alcohol)	24	12.7
Opioid	11	5.8
Cathinone	9	4.8
Methaqualone	2	1.1%
Benzodiazepine	2	1.1%
Inhalant	1	0.5%
Cocaine	1	0.5%
Unknown	1	0.5%

Note: Substances used = *P* < 0.001; Pearson’s Chi-squared tests; the *P*-value is statistically significant.

### Comparison of patients who use substances and those who do not use substances

**FIGURE 1:**
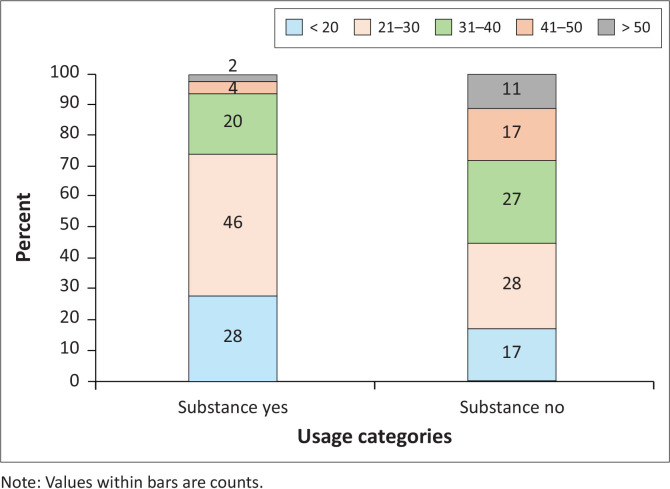
Percentage of patients in the study who used (substance yes) or did not use (substance no) substances by age class.

There was a significant difference between the proportion of patients using substances by age category (*χ*^2^ = 22.39, degrees of freedom [*df*] = 4, *P* = 0.002) (Figure 1). Pairwise analyses using Fisher’s exact tests indicated that younger patients were more likely to use substances, while older patients (i.e. 41–50 years, > 50 years) were more likely to be non-substance users.

**FIGURE 2:**
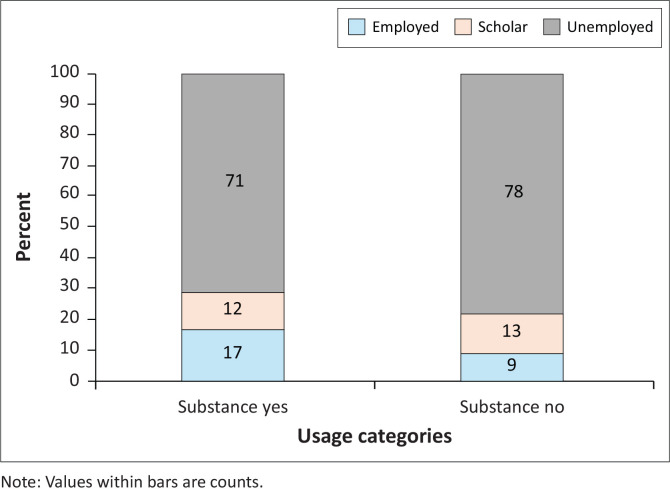
Percentage of patients in the study who used (substance yes) or did not use (substance no) substances by employment status.

There was no significant difference between the proportion of patients using substances and those that did not use substances with regard to employment status (*χ*^2^ = 2.83, *df* = 2, *p* = 0.242) (Figure 2). In both groups, more patients were unemployed than employed or scholars (Figure 2).

**FIGURE 3:**
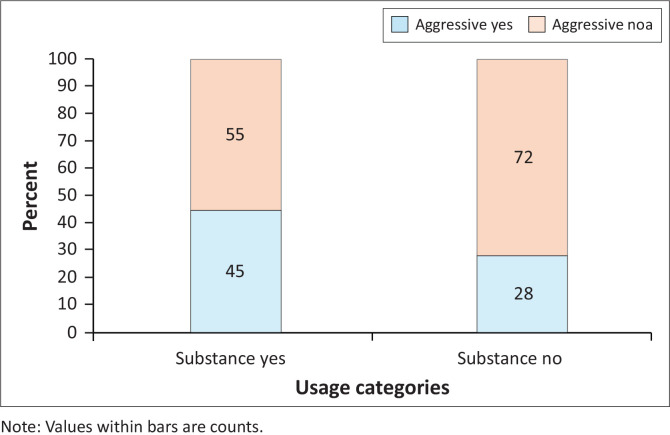
Percentage of patients in the study who used (substance yes) or did not use (substance no) substances that were aggressive or not.

There was a significant difference between the proportion of patients using substances and those who were aggressive (Fisher’s exact test; *P* = 0.019) (Figure 3). A greater proportion of patients using substances were aggressive than those not using substances (Figure 3).

**FIGURE 4:**
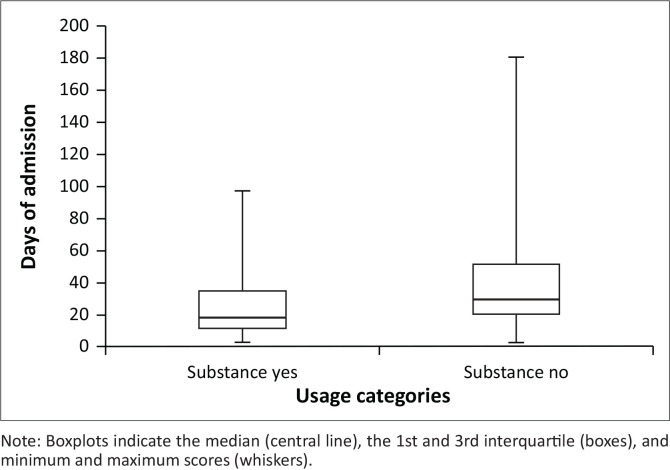
The length of hospital stay in patients who used (substance yes) or did not use (substance no) substances.

Patients who used substances had a significantly shorter length of hospital stay (median = 18 days) compared to patients who did not use substances (median = 29 days) (*Z* score = 4.19, *P* < 0.001) (Figure 4).

## Discussion

### Prevalence of substance use

In this study, the prevalence of substance use among patients presenting with FEP was 73.6%. This high prevalence emphasises the close relationship between substance use and psychosis. The 73.6% prevalence rate of this study is higher than the 55.0%^26^ prevalence rate found in a study done in Cape Town with patients who have psychotic disorders, but lower than the 81.0%^12^ prevalence rate which was reported in a study done in the Eastern Cape which looked at substance use in patients with FEP.

The study done in Cape Town^26^ looked at a broader group of patients, including individuals with a chronic disorder whose substance use patterns may differ from patients with FEP. However, this study and the one conducted in the Eastern Cape^12^ primarily looked at FEP patients, which could possibly explain the similar rates. The slightly lower rates could be explained by differences in regions, population and substance availability. This emphasises the importance of targeted comprehensive assessments and integrated treatment approaches. In addition, it also shows the importance of considering the demographics and social factors when creating strategies for prevention and treating substance use in patients with psychotic disorders.

### Demographic and clinical characteristics of patients presenting with first-episode psychosis

Most of the patients (63%) were male and were largely young people aged 21 years to 30 years. This is consistent with previous research suggesting that FEP patients are predominantly male^12,13,14,15^ and usually have an earlier age of onset.^15,17^

A total of 83.5% of the patients were single, while 15.0% were married or in a long-term relationship and 1.5% were divorced. A study that looked at predictors of schizophrenia in FEP showed a similarly high proportion of single marital status, with 89.0% of their population being single.^27^ This high rate of single marital status could be because FEP commonly occurs in adolescence and early adulthood,^14,16^ which is when individuals often form relationships. Psychosis at this stage has the potential to disrupt this milestone. In addition, negative symptoms such as social withdrawal and a lack of motivation might make it difficult to initiate and maintain these relationships.

Some studies have suggested that being single can have a negative impact on the mental well-being of an individual.^28,29^ Being single is associated with poor social support, and such individuals have reported a low emotional well-being.^29^ Social support is very important in the recovery process of patients with FEP.^30^ Studies have shown that poor social support is associated with poorer outcomes in FEP,^30,31^ including worsening of symptoms and reduced treatment adherence, thus causing higher relapse rates.^32^ Single marital status has been linked to substance use, which can trigger or worsen psychotic symptoms.^32,33^

One of the reasons for hospitalisation was aggressive behaviour, which was present in 36.5% of the patients. The study looked at physical aggression towards others, verbal aggression and property damage as indicators of aggression. Research on violence in patients with FEP in South Africa is not readily available. However, some international studies do provide valuable information. For instance, one study that looked at aggression in FEP patients had a similar level of aggression to our study, with a level of aggression of 34.5%.^34^ A systematic and meta-analysis review published in 2024, which reviewed the level of violence in the past 10 years, reported a lower level (13.4%)^35^ of violence in patients with FEP.

A study conducted in Gauteng province by Anic and Robertson,^14^ which studied a broader clinical population, found that in patients admitted as involuntary users to an acute psychiatric ward, 87.5% had used substances, suggesting that substance use may play a role in the presentation of aggression in psychiatric admissions. Aggression was not stated as a reason for involuntary admission, but it is likely that aggressive behaviour was a contributing factor^14^ to admitting them as involuntary users.

The median length of hospital stay in this study was 24 days. Studies focusing on the length of stay in patients with FEP are scarce. This study specifically focused on FEP patients, and our comparative studies looked at general psychiatric admissions. A study done in KwaZulu-Natal (KZN)^36^ reported a lengthier length of hospital stay of 73 days, and psychotic disorders were identified as a factor associated with a longer duration of hospital stay. Ethiopia^37^ and Brazil^38^ had a median length of stay of 22 days and 25 days, respectively, which are similar to our findings. The Ethiopian study noted that psychotic disorders had the longest hospital stays, with a mean of 34.2 days.^37^ Although studies that look at the length of stay in FEP patients remain scarce, the KZN and Ethiopian studies emphasise the influence psychotic disorders have on prolonging hospital stays.

Social workers play an important role in psychiatric care by providing psychosocial support.^39^ They addresses not only immediate and long-term psychosocial interventions but overall well-being and help patients maintain their functioning socially.^39^ In this study, a vast majority of patients (74%) were not referred to a social worker, revealing a potential gap in holistic care. The low referral rate reported in this study signifies the underutilisation of social services in the management of FEP. The significance of social service integration in the management of early psychosis was highlighted in a study conducted by McGorry et al.^40^ The findings in this study reveal that many patients with FEP did not receive access to community-based intervention and support in their communities. Moreover, these findings are concerning as some of these patients were substance users. Social workers also assist with referring patients to specialised, individualised rehabilitation facilities.^41^

Substance-induced psychotic disorder was the most common diagnosis in this study, affecting 40.0% of patients, followed by schizophrenia (23.5%) and psychotic disorders because of AMC (18.0%). Thungana et al.^12^ reported similar findings. In their study, SIPD was the common psychiatric diagnosis (43.6%), followed by primary psychotic disorders (25.6%). Notably, a study done in Australia^42^ had a slightly higher rate of SIPD (56.0%) among patients with FEP. These findings correspond to previous studies that have shown the significant effects of substance use in FEP.^5,12,13^ According to other research, drugs such as alcohol, cannabis and amphetamines can cause psychotic symptoms,^5,8,43^ which can occur acutely during intoxication and/or withdrawal and can also occur in the longer term, where the symptoms are ongoing despite no recent use of a substance.^44^

It is important to note the possibility of a diagnostic bias in our results. When patients present with both psychotic symptoms and substance use, clinicians are more likely to diagnose SIPD rather than diagnosing schizophrenia which is a more chronic diagnosis.^45^ This bias could lead to underdiagnosing primary psychotic disorders, such as schizophrenia, altering the diagnostic distribution shown in FEP studies.

A significant portion of this population had no medical comorbidities (66.8%), suggesting that a substantial proportion of this population may present with good physical health at the onset of psychosis. This is consistent with the concept that FEP frequently commonly manifests in younger people who are less likely to have chronic illnesses. However, the occurrence of blood-borne infections (20.7%) in patients of this study is notable and needs further attention. In this study, blood-borne infections encompass human immunodeficiency virus (HIV), syphilis and Hepatitis C. A study conducted by Mashapu et al.^46^ reported a 23.8% seroprevalence of HIV among FEP patients, who had no history of substance use. Although the incidence of infections could be linked to lifestyle factors such as risky behaviours associated with using substances,^47^ the findings by Mashapu et al.^46^ suggest that there are other vulnerability factors that may contribute to FEP patients being infected with blood-borne infections.

### Substance use patterns and most commonly used substances

Cannabis was the most used substance in this study, accounting for 46.0%. This was followed by methamphetamine (27.0%) and ethanol (12.7%). These findings are consistent with the South African Community Epidemiology Network on Drug Use (SACENDU)^48^ project January to June 2024 report, which found cannabis (29.0%) as the most used substance in Gauteng province. This was followed by methamphetamine (24.0%) and opioids (19.0%). Thungana et al.^12^ also noted that cannabis (59.8%) was the most used substance, followed by alcohol (57.3%) and stimulants (46.4%), which showed a similar trend as this study. Franken et al.^10^ also reported similar trends in the Western Cape; 51.0% of individuals had used cannabis, and 36.0% had used methamphetamines.

Both cannabis and methamphetamine were prevalent in this study, and both are associated with the development of or worsening of psychosis.^10,12,13^ Methamphetamine is associated with psychotic symptoms and severe behavioural problems, suggesting that it may have a role in the clinical presentation of psychosis in this population.^40,41^

A total of 59% of substance users reported using more than one substance. The SACENDU^49^ project reported a similarly high rate of poly-substance use, with a 56% rate in South Africa and a 58% rate in Gauteng province. These findings suggest that poly-substance use is quite common and is a nationwide issue that needs integrated strategies that can handle substance combinations.

### Comparison of clinical and demographic characteristics between substance users and non-users

Substance use was significantly higher in the younger population than in the older population. Only 4% of individuals aged 41–50 years and 2% above the age of 50 years reported using substances, whereas 43% of patients between the ages of 21 years and 30 years and 28% under the age of 20 years had used substances. The SACENDU^48^ project reported that 84% of cannabis users in Gauteng province were less than 18 years of age. We also need to be cognisant of the role peer pressure can have on adolescence and early adulthood. Research has shown that peer pressure does play a significant role in adolescent and young adults using substances.^50,51^

The study did not find a significant difference when comparing substance users to non-users in terms of employment status. More people were unemployed than employed or scholars in both groups. A study in done in Johannesburg had similar findings, where there was no difference in employment status when comparing substance users with non-users.^14^ These findings highlight that, independent of substance use, unemployment is a common characteristic in patients with psychosis, as demonstrated by Ramsay et al.^52^ This study was conducted in Soweto, a region that has high overall unemployment rates.^23^ This background emphasises how crucial it is to address socioeconomic factors such as job scarcity when developing treatment strategies for FEP. Employment support programmes could improve mental health outcomes in this population.

Substance use was closely related to aggressive behaviour, with 45% of patients who used substances presenting with a history of aggression prior to admission compared to 28% of non-users. Anic and Robertson^14^ reported that among the patients they had admitted to their acute ward, 80% of their population had presented with psychotic symptoms and 50% had presented with aggressive behaviour. While a study done by Weich and Pienaar^16^ reported 59% of their patients that had used substances were aggressive versus 26% of non-users. The study done by Weich and Pienaar analysed general psychiatric admissions. However, their results do suggest that the use of substances does play a role in the presentation of aggression. Patients who have used substances are most likely to be brought in by police or ambulance officials because of their aggressive behaviour.^10,12,14,16^

Although the median duration of hospital stay was lower (18 days) for substance users when compared to 29 days of non-users, this may not always mean that their outcomes were better. Substance use magnifies psychotic symptoms,^10,12,13,15^ and treatment usually focuses on removing the substance and managing the acute symptoms,^53^ and the symptoms usually respond within 30 days,^54^ meaning a faster resolution of symptoms.^53,54^ Shorter hospital stays, however, may in addition hinder access to holistic care which could increase readmission rates. Substance users are at increased risk of readmissions and overall poor clinical outcomes.^55^ This emphasises the need for integrated approaches when managing patients with FEP with substance use.

### Implications

The study highlights key socio-demographic and clinical factors associated with drug use among patients admitted to CHBAH. The significant role of substance abuse, mainly cannabis and methamphetamines, in the triggering and aggravating of psychotic symptoms underscores the need for targeted early intervention programmes, particularly for disadvantaged young people. In addition, the study highlights gaps in integrated care, including the underutilisation of social work services that can be addressed by integrated care models, combined with mental health and drug abuse services. This study did not include psychology and occupational therapy. This was a conscious decision to narrow the variables and include factors that are most pertinent to substance use in FEP. This exclusion does not imply that psychology and occupational therapy are not significant. They are a crucial component of the multidisciplinary care in patients with FEP and substance use, and future studies may delve deeper into the role of these disciplines.

### Limitations

The study is limited by its retrospective design based on existing records. Medical records that had missing information were excluded which caused selection bias. In addition, as this was a retrospective study, the data that were in the records could not be independently verified for accuracy, introducing possible information bias. Samples were collected from a single hospital in a socioeconomically disadvantaged region, which may limit the generalisation of the results to other areas and populations. This study did not look at occupational therapy and psychology referrals, which are important components of the multidisciplinary approach in the management of FEP and substance use. This exclusion limits the ability to comment on the full multidisciplinary team approach to FEP and substance use. Moreover, the use of self-reported or historical records of substance use may underestimate the prevalence of substance use because of a lack of reporting or inconsistent testing. This study looked at the lifetime history of substance use and not only at current use of substances which could contribute to bias.

## Conclusion

The study emphasises the critical impact of the use of substances on the clinical and socio-demographic characteristics of patients with FEP, which highlights that cannabis and methamphetamines are associated with the emergence of a psychotic disorder. Younger age, male gender and aggressive behaviours are significantly related to substance use and emphasise the need for targeted early intervention programmes and gender-sensitive approaches. The findings also reveal deficiencies in holistic care, including insufficient referrals to social workers, and point to the need for integrated care models that address both psychiatric and substance use disorders. These insights can guide effective prevention, treatment and policy-making interventions to reduce the dual burden of mental illness and substance use, and ultimately improve the outcomes and health systems of patients and resource-limited settings.

## References

[1] National Alliance on Mental Illness. Early psychosis and psychosis [homepage on the Internet]. [cited 2023 Jun 16]. Available from: https://www.nami.org/About-Mental-Illness/Mental-Health-Conditions/Psychosis

[2] American Psychiatric Association. Diagnostic and statistical manual of mental disorders. 5th ed. Arlington, VA: American Psychiatric Publishing; 2013.

[3] Breitford NJK, Srihari VH, Woods SW. Review of the operational definition for first episode psychosis. Early Interv Psychiatry. 2009;3(4):259–265. 10.1111/j.1751-7893.2009.00148.x 22642728 PMC4451818

[4] Bromley S, Choi M, Faruqui S. First episode psychosis – An information guide. Rev. ed. Centre for Addiction and Mental Health; 2015.

[5] DiForti M, Quattrone D, Freeman TP, et al. The contribution of cannabis use to variation in the incidence of psychotic disorder across Europe (EU-GEI): A multicenter case-control study. Lancet Psychiatry. 2019;6(5):427–436. 10.1016/S2215-0366(19)30048-3 30902669 PMC7646282

[6] Wang J, Kapoor M, Goate AM. The genetics of substance dependence. Ann Rev Genomics Hum Genet. 2012;13:241–261. 10.1146/annurev-genom-090711-163844 22703173 PMC3474605

[7] Green AI, Khokhar JY. Addiction and schizophrenia: A translational perspective. Schizophr Res. 2018;194:1–3. 10.1016/j.schres.2017.10.008 29046253

[8] Barnett JH, Werners U, Secher SM, et al. Substance use in a population-based clinic sample of people with first episode psychosis. Br J Psychiatry. 2007;190(6):515–520. 10.1192/bjp.bp.106.024448 17541112

[9] World Health Organization. Drugs (psychoactive) [Fact Sheet] [homepage on the Internet]. Geneva: World Health Organization [cited 2025 Jan 07]. Available from: https://www.who.int/health-topics/drugs-psychoactive

[10] Franken H, Parker J, Allen R, et al. A profile of adult acute admissions to Lentegeur Psychiatric Hospital, South Africa. S Afr J Psychiatry. 2019;25:a1244. 10.4102/sajpsychiatry.v25i0.1244 PMC6779967 31616578

[11] Sukeri K. Regional aspects of long term-public sector psychiatric care in the Eastern Cape. S Afr J Psychiatr. 2017;23:a6. 10.4102/sajpsychiatry.v23i0.992 PMC6138187 30263179

[12] Thungana Y, Zingela Z, Van Wyk S. First-episode psychosis and substance use in Nelson Mandela Bay: Findings from an acute mental health unit. S Afr J Psychiatr. 2019;25:a1372. 10.4102/sajpsychiatry.v25i0.1372 PMC6851873 31745443

[13] Wade D, Harrigan S, Edwards S. Substance misuse in first-episode psychosis: 15-month prospective follow-up study. Br J Psychiatry. 2006;189(3):229–234. 10.1192/bjp.bp.105.017236 16946357

[14] Anic A, Roberston L. Substance use among acute psychiatric inpatients, Gauteng, South Africa. S Afr J Psychiatr. 2020;26:a1526. 10.4102/sajpsychiatry.v26i0.1526 PMC7565019 33101728

[15] Inchausti L, Gorostiza I, Gonzalez Torres MA, et al. Diagnostic stability in substance-induced psychosis. Rev Psiquiatr Salud Ment. 2022;15(4):272–280. 10.1016/j.rpsm.2019.10.005 36400700

[16] Weich L, Pienaar W. Occurrence of comorbid substance use disorders among acute psychiatric inpatients at Stikland Hospital in the Western Cape, South Africa. Afr J Psychiatry. 2009;12(3):213–217. 10.4314/ajpsy.v12i3.48496 19750250

[17] Hodgins S, Mednick SA, Brennan PA, et al. Mental Disorders and crime. Evidence from a Danish birth cohort. Arch Gen Psychiatry. 1996;53(6):489–496. 10.1001/archpsyc.1996.01830060031004 8639031

[18] South African Government. National Drug Master Plan 2019 to 2024 [homepage on the Internet]. Pretoria: Department of Social Development; 2020 [cited 2022 Apr 10]. Available from: https://www.gov.za/sites/default/files/gcis_document/202006/drug-master-plan.pdf

[19] Nguse S, Wassenaar D. Mental health and COVID-19 in South Africa. S Afr J Psychol. 2021;51(2):304–313. 10.1177/00812463211001543 38603189 PMC8107260

[20] Tarricone I, Boydell J, Panigada S, et al. The impact of substance use at psychosis onset on First Episode Psychosis course: Results from a 1-year follow-up study in Bologna. Schizophr Res. 2014;156(1):60–63. 10.1016/j.schres.2014.01.014 24525084

[21] Mousavi SB, Higgs P, Piri N, et al. Prevalence of substance use among psychotic patients and determining its strongest predictor. Iran J Psychiatry. 2021;16(2):124–130. 10.18502/ijps.v16i2.5812 34221037 PMC8233556

[22] Vassar M, Holzmann M. The retrospective chart review: Important methodological considerations. J Educ Eval Health Prof. 2013;10:12. 10.3352/jeehp.2013.10.12 24324853 PMC3853868

[23] Republic of South Africa, Department of Cooperative Governance and Traditional Affairs. City of Johannesburg Profile – October 2020 [homepage on the Internet]. Pretoria: Department of Cooperative Governance and Traditional Affairs; 2020 [cited 2025 Sep 03]. Available from: https://www.cogta.gov.za/ddm/wp-content/uploads/2020/11/City-of-Johannesburg-October-2020.pdf

[24] Chris Hani Baragwanath Academic Hospital. Psychiatry Department [homepage on the Internet]. Johannesburg: Chris Hani Baragwanath Hospital; 2025 [cited 2024 Dec 18]. Available from: https://www.chrishanibaragwanathhospital.co.za/departments/psychiatry/show

[25] Ramchander P. Chapter 2: Background to the study area: Soweto. In: Towards the responsible management of the socio-cultural impact of township tourism [dissertation on the internet]. Pretoria: University of Pretoria; 2004 [cited 2024 Jan 07]. Available from: https://repository.up.ac.za/bitstream/handle/2263/27544/02chapter2.pdf?sequence=3&isAllowed=y

[26] Temmingh HS, Mall S, Howells FM, et al. The prevalence and clinical correlates of substance use disorders in patients with psychotic disorder from an Upper-Middle-Income Country. S Afr J Psychiatr. 2020;26:a1473. 10.4102/sajpsychiatry.v26i0.1473 PMC7433243 32832129

[27] Ramirez N, Arran B, Salavert J, et al. Predictors of schizophrenia in patients with a first episode of psychosis. Psychiatry Res. 2010;175(1–2):11–14. 10.1016/j.psychres.2009.03.013 19923008

[28] Apostolou M, Alexopoulos S, Christoforou C. The price of being single: An explorative study of the disadvantages of singlehood. Pers Individ Dif. 2023;208:112208.

[29] Adamczyk K, Segrin C. Perceived social support and mental health among single vs partnered Polish young adults. Curr Psychol. 2015;34(1):82–96. 10.1007/s12144-014-9242-5 25774079 PMC4348549

[30] Serra-Arumi C, Vila-Badia R, Del Cacho N, et al. Association of perceived social support with sociodemographic, clinical, and psychosocial variables in patients with first episode psychosis. Psychiatry Res. 2023;162:30–36. 10.1016/j.jpsychires.2023.04.008 37075638

[31] Gayer-Anderson C, Morgan C. Social support and three-year symptom and admission outcomes for first episode psychosis. Schizophr Res. 2006;80(2–3):227–234. 10.1016/j.schres.2005.05.006 15964175

[32] Flemming CB, White HR, Oesterle S, et al. Romantic relationship status changes and substance use among 18- to-20- year-olds. J Stud Alcohol Drugs. 2010;71(6):847–856. 20946741 10.15288/jsad.2010.71.847 PMC2965298

[33] Jang BJ, Schuler MS, Evans-Polce RJ, et al. Marital status as partial mediator of the associations between young adult substance use and subsequent substance use disorder: Applications of causal inference methods. J Stud Alcohol Drugs. 2018;79(4):567–577. 10.15288/jsad.2018.79.567 30079872 PMC6090100

[34] Large M, Nielssen O. Violence in first-episode psychosis: A systematic review and meta-analysis. Schizophr Res. 2011;125(2–3):209–220. 10.1016/j.schres.2010.11.026 21208783

[35] Youn S, Guadagno BL, Byrne LK, et al. Systematic review and meta-analysis: Rates of violence during First Episode Psychosis (FEP). Schizophr Bull. 2024;50(4):757–770. 10.1093/schbul/sbae010 38412435 PMC11283196

[36] Myeni SY, Ntlantsana V, Tomita A, et al. The profile of long -stay patients in a psychiatric hospital in KwaZulu-Natal, South Africa. S Afr J Psychiatr. 2025;31:a2358. 10.4102/sajpsychiatry.v31i0.2358 PMC11830845 39968315

[37] Addisu F, Wondafrash M, Chemali Z, et al. Length of stay of psychiatric admissions in a general hospital in Ethiopia: A retrospective study. Int J Ment Health Syst. 2015;9:13. 10.1186/s13033-015-0006-x 25780386 PMC4361196

[38] Baeza FL, Da Rocha NS, Fleck MP. Predictors of length of stay in an acute psychiatric inpatient facility in a general hospital: A prospective study. Braz J Psychiatry. 2017;40(1):89–96. 28700014 10.1590/1516-4446-2016-2155 PMC6899424

[39] McNamara KA. Professionals and their organizational roles in pathways to care for early psychosis: Where are the social workers? [disser$4tation on the Internet]. Baltimore, MD: University of Maryland, Baltimore; 2014 [cited 2025 Mar 10]. Available from: https://www.proquest.com/dissertations-theses/professionals-their-organizational-roles-pathways/docview/1545891885/se-2?accountid=15083

[40] McGorry PD, Killackey E, Yung A. Early intervention in psychosis: Concepts, evidence, and future directions. Med J Aust. 2007;187(S7):S26–S30. 10.5694/j.1326-5377.2007.tb01327.x 17908033

[41] Lukens EP, McFarlane WR. Psychoeducation as evidence-based practice: Consideration for practice, research, and policy. Br Treat Crisis Interv. 2004:4(1);205–225. 10.1093/brief-treatment/mhh019

[42] Fraser S, Hides L, Philips L, Proctor D, Lubman DI. Differentiating first episode substance induced and primary psychotic disorders with concurrent substance use in young people. Schizophr Res. 2012;136(1–3):110–115. 10.1016/j.schres.2012.01.022 22321667

[43] Hunt GE, Large MM, Cleary M, et al. Prevalence of comorbid substance use in schizophrenia spectrum disorders in community and clinical settings, 1990–2017: Systematic review and meta-analysis. Drug Alcohol Depend. 2018;191:234–258. 10.1016/j.drugalcdep.2018.07.011 30153606

[44] Stefanis NC, Dragovic M, Power BD, et al. The effect of drug use on the age at onset of psychotic disorders in an Australian cohort. Schizophr Res. 2014;156(2):211–216. 24831390 10.1016/j.schres.2014.04.003

[45] Caton CLM, Samet S, Hasin DS. When acute-state psychosis and substance use co-occur: Differentiating substance-induced and primary psychotic disorders. J Psychiatr Pract. 2000;6(5):256–266. 10.1097/00131746-200009000-00006 15990489

[46] Mashapu S, Mkhize DL. HIV seropositivity in patients with first episode psychosis. S Afr J Psychiatr. 2007;13(3):a2.

[47] Brown E, Castagnini E, Langstone A, et al. High-risk sexual behaviours in young people experiencing a first episode of psychosis. Early Interv Psychiatry. 2023;17(2):159–166. 10.1111/eip.13301 35355426

[48] SACENDU. SACENDU UPDATE: January–June 2024 [homepage on the Internet]. Cape Town: South African Medical Research Council; 2024 [cited 2025 Mar 11]. Available from: https://www.samrc.ac.za/sites/default/files/attachments/2025-02/SacenduUpdateJanJun2024.pdf

[49] South African Medical Research Council. SACENDU research brief: Monitoring alcohol, tobacco, and other drug use trends in South Africa (Phase 55) [homepage on the Internet]. 2025 [cited 2025 Mar 11]. Available from: https://www.samrc.ac.za/sites/default/files/attachments/2025-02/SACENDUBriefPhase55.pdf

[50] Keyzers A, Lee SK, Dworkin J. Peer pressure and substance use in emerging adulthood: A latent profile analysis. Subst Use Misuse. 2020;55(10):1716–1723. 10.1080/10826084.2020.1759642 32400279

[51] Watts LL, Hamza EA, Bedewy DA, et al. A meta-analysis study on peer influence and adolescent substance use. Curr Psychol. 2024;43:3866–3881. 10.1007/s12144-023-04944-z

[52] Ramsay CE, Stewart T, Compton MT. Unemployment among patients with newly diagnosed first-episode psychosis: Prevalence and clinical correlates in a U.S sample. Soc Psychiatry Psychiatr Epidemiol. 2012;47(5):797–803. 10.1007/s00127-011-0386-4 21541697

[53] Baldacara L, Ramos A, Castaldelli-Maia JM. Managing drug-induced psychosis. Int Rev Psychiatry. 2023;35(5–6):496–502. 10.1080/09540261.2023.2261544 38299647

[54] Ries RK, Russo J, Wingerson D, et al. Shorter hospital stays and more rapid improvement among patients with schizophrenia and substance disorders. Psychiatr Serv. 2000;51(2):210–215. 10.1176/appi.ps.51.2.210 10655005

[55] Burrer A, Egger ST, Spiller TR, et al. Examining the impact of substance use on hospital length of stay in schizophrenia spectrum disorder: A retrospective analysis. BMC Med. 2024;22:233. 10.1186/s12916-024-03447-3 38853281 PMC11163832

